# Morphological and colour morph clines along an altitudinal gradient in the meadow grasshopper *Pseudochorthippus parallelus*

**DOI:** 10.1371/journal.pone.0189815

**Published:** 2017-12-28

**Authors:** Günter Köhler, Jörg Samietz, Holger Schielzeth

**Affiliations:** Population Ecology Group, Institute of Ecology, Friedrich Schiller University, Jena, Germany; Universidad de Granada, SPAIN

## Abstract

Many animals show altitudinal clines in size, shape and body colour. Increases in body size and reduction in the length of body appendices in colder habitats are usually attributed to improved heat conservation at lower surface-to-volume ratios (known as Bergmann’s and Allen’s rule, respectively). However, the patterns are more variable and sometimes reversed in small ectotherms that are affected by shortened growing seasons. Altitude can also affect colouration. The thermal melanism hypothesis predicts darker colours under cooler conditions because of a thermoregulatory advantage. Darker colours may also be favoured at high altitudes for reasons of UV protection or habitat-dependent crypsis. We studied altitudinal variation in morphology and colour in the colour-polymorphic meadow grasshopper *Pseudochorthippus parallelus* based on 563 individuals from 17 populations sampled between 450 and 2,500 m asl. Pronotum length did not change with altitude, while postfemur length decreased significantly in both sexes. Tegmen (forewing) length decreased in males, but not in females. The results indicate that while body size, as best quantified by pronotum length, was remarkably constant, extended appendices were reduced at high altitudes. The pattern thus follows Allen’s rule, but neither Bergmann’s nor converse Bergmann’s rule. These results indicate that inference of converse Bergmann’s rule based on measurements from appendices should be treated with some caution. Colour morph ratios showed significant changes in both sexes from lowland populations dominated by green individuals to high-altitude populations dominated by brown ones. The increase of brown morphs was particularly steep between 1,500 and 2,000 m asl. The results suggest shared control of colour in males and females and local adaptation along the altitudinal gradient following the predictions of the thermal melanism hypothesis. Interestingly, both patterns, the reduction of body appendices and the higher frequency of brown individuals, may be explained by a need for efficient thermoregulation under high-altitude conditions.

## Introduction

High-altitude habitats are characterized by extreme conditions and animals that inhabit them often differ phenotypically from lowland populations [1]. Altitudinal gradients are typically very extreme, for example with respect to changes in temperature (5.5 K per 1,000 m in altitude, [2]), amount and intensity of solar radiation, and duration of the vegetation season. The steepness of these gradients makes altitudinal variation particularly relevant for analysing phenotypic clines in widespread species that occur from low to high altitudes [2]. Systematic changes in morphology and body colour have been described (e.g. [3–7]), but at least for small ectothermic species the patterns are rather variable and the ultimate causes of such phenotypic clines are debated [8, 9].

It has long been recognized that animals tend to increase in body size and decrease the size of their appendices in colder climates. These two geographic patterns are known as Bergmann’s rule [3] and Allen’s rule [4], respectively. The original publications refer to latitudinal gradients only, but the two rules are now typically discussed more broadly, covering latitudinal and altitudinal changes. The standard explanation for both rules rest on the argument that high volume-to-surface ratios help in reducing convective heat loss. The original formulations of Bergmann’s as well as Allen’s rule were based only on endothermic species. But even a number of ectothermic insects show latitudinal and/or altitudinal Bergmann clines [5, 6, 9] and there is also some evidence for a reduction in the length of appendices in insects [5]. However, convective heat loss may thus be less critical in ectothermic insects, because insects typically do not use metabolic heat for maintaining body temperature and thus do not lose acquired resources by convective cooling. Hence the causes of morphological clines in ectotherms may differ from the causes that apply to endotherms. For example, it has been speculated that even ectothermic species follow Bergmann’s rule because of increased cell sizes at lower temperatures [10].

However, there are also reasons that act in the opposite direction and result in patterns that have been described as converse Bergmann’s rule [9]. In ectothermic species, large surface relative to volume may actually facilitate behavioural heating from solar radiation, and this may or may not compensate for faster heat loss [11]. Furthermore, body size in ectothermic insects can be limited by the duration of the growing season and this may limit final body sizes [9]. Consequently, the patterns are more variable in ectotherms [6, 12, 9]. Interestingly, cases of Bergmann’s clines in connection with slower growth in insects seem to be genuinely related to the effect of temperature per se rather than stressful conditions in general, because slow growth caused by other stressors such as food limitation results in smaller rather than larger body sizes [5, 8, 13].

Altitudinal gradients can also affect body colour. The thermal melanism hypothesis, for example, predicts that individuals in cooler climates, i.e. higher latitudes or altitudes, are darker than individuals from warmer climates because of more efficient use of limited solar radiation for thermoregulation [7]. This can be of significant importance when solar radiation is limiting, for example because it allows a larger time window for daily activities [7]. Darker colours at higher altitudes may also be favoured for reasons of UV protection, even though this does not very well explain clines of increased darkness at higher latitudes. Furthermore, habitats typically change with altitude and this can affect the cryptic value of different body colours, which may favour dark colours in ground-dwelling prey species. However, habitat-dependent changes are likely to have a more variable effect on different species.

We here investigate spatial variation in morphology and colour across a steep altitudinal gradient in the meadow grasshopper *Pseudochorthippus parallelus* (Zetterstedt, 1821) (Acrididae, Gomphocerinae, formerly *Chorthippus parallelus*). The species is one of the most abundant grasshoppers in Europe and occurs across a wide range of grassland habitats. The latitudinal distribution in Europe range between 35°-65° N, from the Pyrenees, Southern Italy and Greece in the South to Fennoscandinavia beyond the polar circle in the North [14]. Its wide altitudinal range is also unusual, reaching from sea level up to 2,700 m asl in the Alps [15, 16]. The wide altitudinal range and high local densities make it an exceptionally suitable species among the European Orthoptera for studying altitudinal gradients.

*P*. *parallelus* is apparently exclusively univoltine with four juvenile instars in both sexes (in females occasionally five) [17, 15]. Like most Gomphocerine grasshoppers, the species exhibits a distinct sexual dimorphism, with females being larger than males. Both sexes are brachypterous with reduced alae (hind wings) and females also possess shortened tegmina (forewings), while tegmina are longer in males and are used for sound production (stridulation). The short alae make both sexes unable to fly. Macropterous (long-winged) individuals occur in low frequencies in most populations and are sometimes capable of flight [18]. *P*. *parallelus* is also remarkable for its colour polymorphism in both sexes. Some individuals are entirely green while others lack green tones altogether. Intermediate morphs can be green with brown legs, brown sides or brown dorsal stripes. All morphs are easily recognised and classified [19] and have been found to be genetically inherited [20, 21]. Morph ratios have been found to be temporarily stable in local populations [19, 22].

Our study is based on extensive series of specimens from the Nadig collection (Switzerland) collected in the 1950s to 1980s from the Swiss and Italian Alps. We aimed to test if the species responds to colder temperatures and a shorter season at higher altitudes by reduced size and more compact stature. We thus expect populations to follow the converse of Bergmann’s rule and the standard version of Allen’s rule [12, 9]. To this end, we measured three morphological traits that gave information on size (pronotum length) and shape (postfemur and tegmen length) of the individuals. Furthermore, we aimed to test if there is evidence for local adaptation in colour morph compositions across the altitudinal gradient. In particular, we expect high-altitude populations to be dominated by darker morphs, in line with the thermal melanism hypothesis that suggests darker colours at high altitudes (and latitudes) due to improved thermoregulation [7].

## Material and methods

### Sample collection, measurements, and colour morphs

Meadow grasshoppers were collected haphazardly in large series by Dr. Adolf Nadig (1910–2003), a recognized Swiss orthopterologist. All specimens were dried and needled and assembled in standard insect boxes. In September 1993, we (GK, JS) got the permission to inspect Dr. Nadig’s extraordinary collection in Chur/Switzerland and we focused on surveying the samples of *P*. *parallelus*. Each series was labelled with a site name and the altitudinal range at which they were collected (we used the midpoint of altitudinal ranges in further analyses). The complete collection of more than 100.000 individuals of around 800 Orthoptera species was donated to the Muséum d´histoire naturelle de Genève in 2001 [23].

Among the available specimens of *P*. *parallelus*, we selected larger series of samples from 17 sites from Switzerland and the Italian Alps. Series were selected such that they covered a wide altitudinal range (430–2,500 m asl) while being confined to a relatively small geographic region in the Central Alps. A gap between 500 and 1,100 m asl was unavoidable due to the lack of suitable series. We measured all available individuals from each site (mean sample size 33 individuals per site, range 18–54, Table 1), in total 563 individuals (339 females, 224 males).

**Table 1:** Sampling sites, sampling dates and sample sizes. Site, altitude and date according the labels in the Nadig collection, coordinates approximated using Google Maps. Abbreviations: CH = Switzerland, FL = Fürstentum Liechtenstein, I = Italy. Sex: F = females, M = males.

Site	Coordinates	Altitude	Sampling date	Sample size
Ruggeller Ried (FL)	47°15.15 N, 9°32.53 E	430 m	11/08/1982	20 F + 10 M
Hudelmoos (S Amriswil, CH)	47°31.25 N, 9°17.13 E	500 m	11/08/1960	30 F + 24 M
Val Gerola above Fenile / Val Tellina (I)	46°03.29 N, 9°32.53 E	1,150–1,350 m	03/09/1973 06/09/1973	18 F + 14 M
Monte Legnone, S-Grat J. (I)	46°04.09 N, 9°24.00 E	1,400–1,600 m	29/08/1974	18 F + 12 M
Locarno, Cardada (CH)	46°11.55 N, 8°47.15 E	1,450–1,600 m	13/09/1959	18 F + 17 M
Ardez, Pradasura (Lower-Engadin—CH)	46°46.50 N, 10°12.10 E	1,580–1,700 m	19/09/1971	15 F + 11 M
Monte Grappa (Venez. Alps—I)	45°52.05 N, 11°47.59 E	1,600–1,750 m	23/08/1973	15 F + 10 M
Locarno, Cardada (CH)	46°52.17 N, 8°47.31 E	1,650–1,800 m	17/07/1959 13/09/1959	14 F + 10 M
Monte Legnone, S-Grat J. (I)	46°04.40 N, 9°24.32 E	1,650–1,850 m	29/08/1974	14 F + 4 M
A. Varuna (Poschiavo) (CH)	46°19.42 N, 10°01.59 E	1,900 m	23/09/1967	20 F + 5 M
Blanca, Grevasalvas (Upper Engadin—CH)	46°25.29 N, 9°42.52 E	1,940–2,080 m	16/10/1971	22 F + 19 M
Ardez, Murtera d´Artez (Lower Engadin—CH)	46°47.08 N, 10°11.54 E	2,100 m	19/09/1971	21 F + 4 M
Kleine Scheidegg (CH)	46°35.11 N, 7°57.32 E	2,050–2,150 m	11/09/1982	20 F + 17 M
Ftan GR, P. Minschun (CH)	46°48.33 N, 10°15.26 E	2,200 m	30/09/1972	30 F + 14 M
Val Malenco, Monte Molta (I)	46°17.17 N, 09°53.06 E	2,280–2,330 m	06/09/1971	22 F + 24 M
Bindelweg, Dolomiten (I)	46°24.10 N, 11°50.19 E	2,380–2,500 m	18/09/1974	18 F + 7 M
Muothas,Muraigl (Engadin-CH)	46°31.22 N, 9°54.17 E	2,500 m	13/09/1951	24 F + 22 M

Morphological traits were measured under a Zeiss stereomicroscope (type SM XX) using an ocular graticule. For each individual we identified the sex and measured pronotum length (at 12,5× magnification), postfemur length on left and right side (7,9× magnification) and tegmen length on both sides (7,9× magnification) following the procedure of Reynolds [24]. Many studies use postfemur length as a proxy of body size in Orthoptera (e.g. [12, 25, 26]), but pronotum length is considered the most reliable indicator of body size, although it is more rarely measured (e.g. [27–29]). Because of nearly identical measurements from the two sides of the body, both in postfemora and in tegmina, we used only the right-side measurements in the analysis (with few exceptions in which we used the measurement of the left postfemur if the right one was missing).

**Fig 1:**
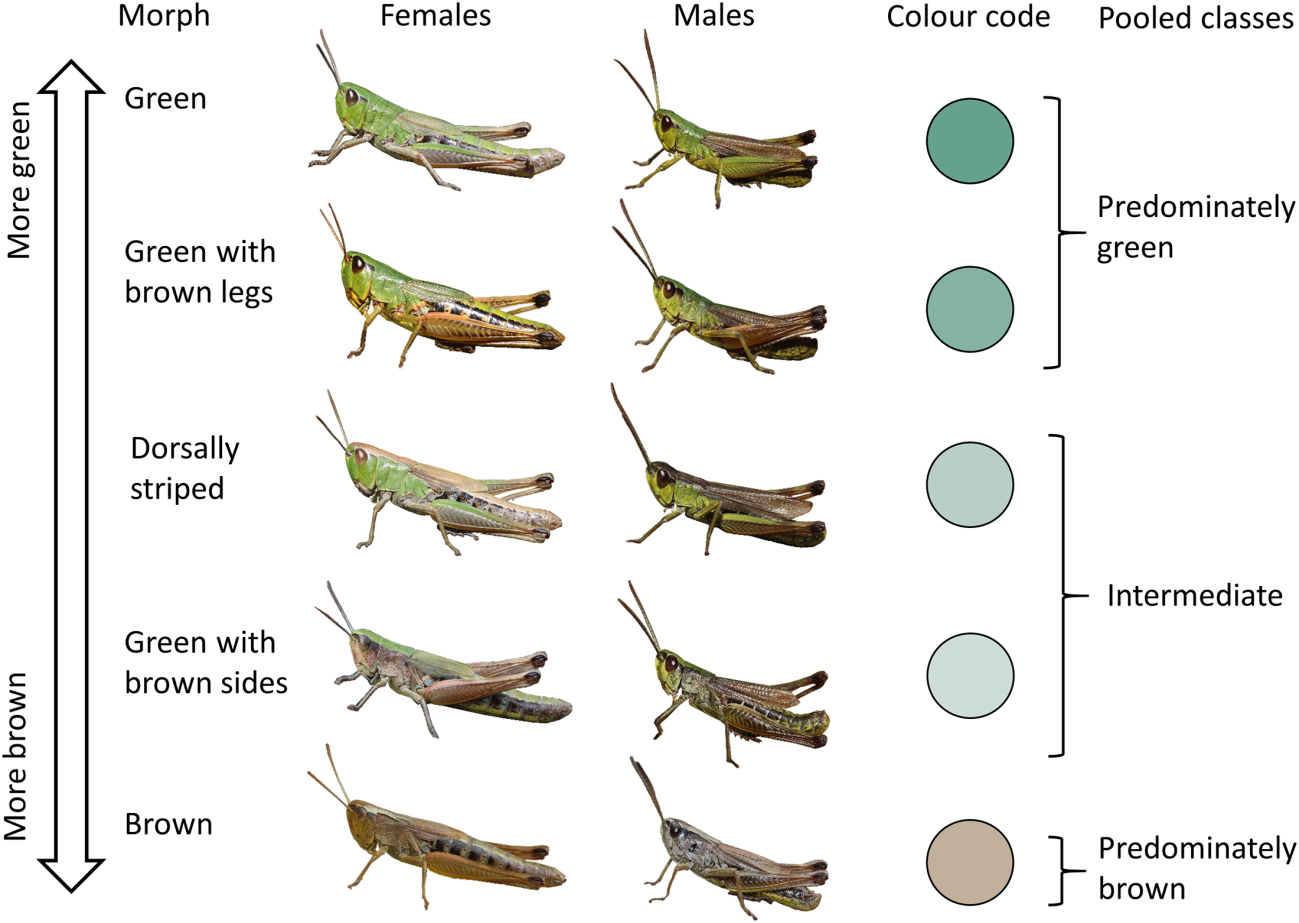
Colour morph classification for both sexes of *Pseudochorthippus parallelus*. Images show individuals photographed alive at other sites, not individuals from the Nadig collection.

We also scored each individual’s colour morph based on a pre-established classification into five distinct morphs [19, 21]: (i) green, (ii) green with brown legs, (iii) brown dorsal stripe, (iv) green with brown sides and (v) completely brown (Fig 1). Although green colours tend to fade, with some experience, colour morph identity is easily recognized even on dried materials. Since we assume that the relative area of visible green and brown parts is most ecologically relevant, we lumped morphs (i)+(ii) as predominately green and morphs (iii)+(iv) as intermediate in contrast to (v) completely brown in the analysis of broad altitudinal trends (Fig 1). However, we keep the original morph classification in the analysis of morphological differences among morphs, since the five morphs appear to be genetically distinct [21].

### Data analysis

We used linear mixed effects models (LMM) with Gaussian error distributions in the analysis of morphological traits and generalized linear mixed effects models (GLMM) with logit link and binomial error distribution in the analysis of colour morphs. Each model included *altitude* as the fixed effect of primary interest. Altitude (measured in meters above sea level) was divided by hundred, such that slopes represent the average change in trait values per 100 m increase in altitude. Furthermore, we included *year of sampling* as a fixed effect to test for potential time trends in the data. All models also included a random effect of *site identity* to control for spatial heterogeneity in phenotypes. In the analysis of morphological differences among morphs, we included *morph identity* as a fixed factor while controlling for altitude, year of sampling and site identity.

Because of the marked sexual size dimorphism, it is well possible that altitudinal gradients and residual variances differ between the sexes and this can violate model assumptions. We therefore analysed the sexes separately in the initial analysis. Slopes in the two sexes are thus estimated based on independent data and from separate models and can be seen as replications of altitudinal trends in size and colour. However, explicit testing for differences between slopes needs to be done in a joint model including *sex* and the *sex-by-altitude interaction* as fixed effects. We therefore provide the estimates for the interaction whenever necessary.

The random effect variance components are frequently neglected when fixed effects are the primary interest of the analysis, even though these variances provide relevant information about the hierarchical structure of phenotypic variation [30]. In our case the random effect variances represent spatial heterogeneity and may result from population history and demography or from environmental variation other than what is captured by altitude. We present random effect components in the form of adjusted repeatabilities *R*, i.e. variance components standardized by the phenotypic variance after controlling for the effect of altitude and year of sampling [31]. Standard errors of repeatabilities were quantified by parametric bootstrapping with 10,000 iterations.

All analyses were performed in R 3.3.2 [32] using the package lme4 1.1–12 [33] for model fitting, the package lmerTest 2.0–33 [34] for significance testing of fixed effects and the package rptR 0.9.2 [35] for estimating ratios of variance components and their standard errors.

## Results

### Morphology

**Fig 2:**
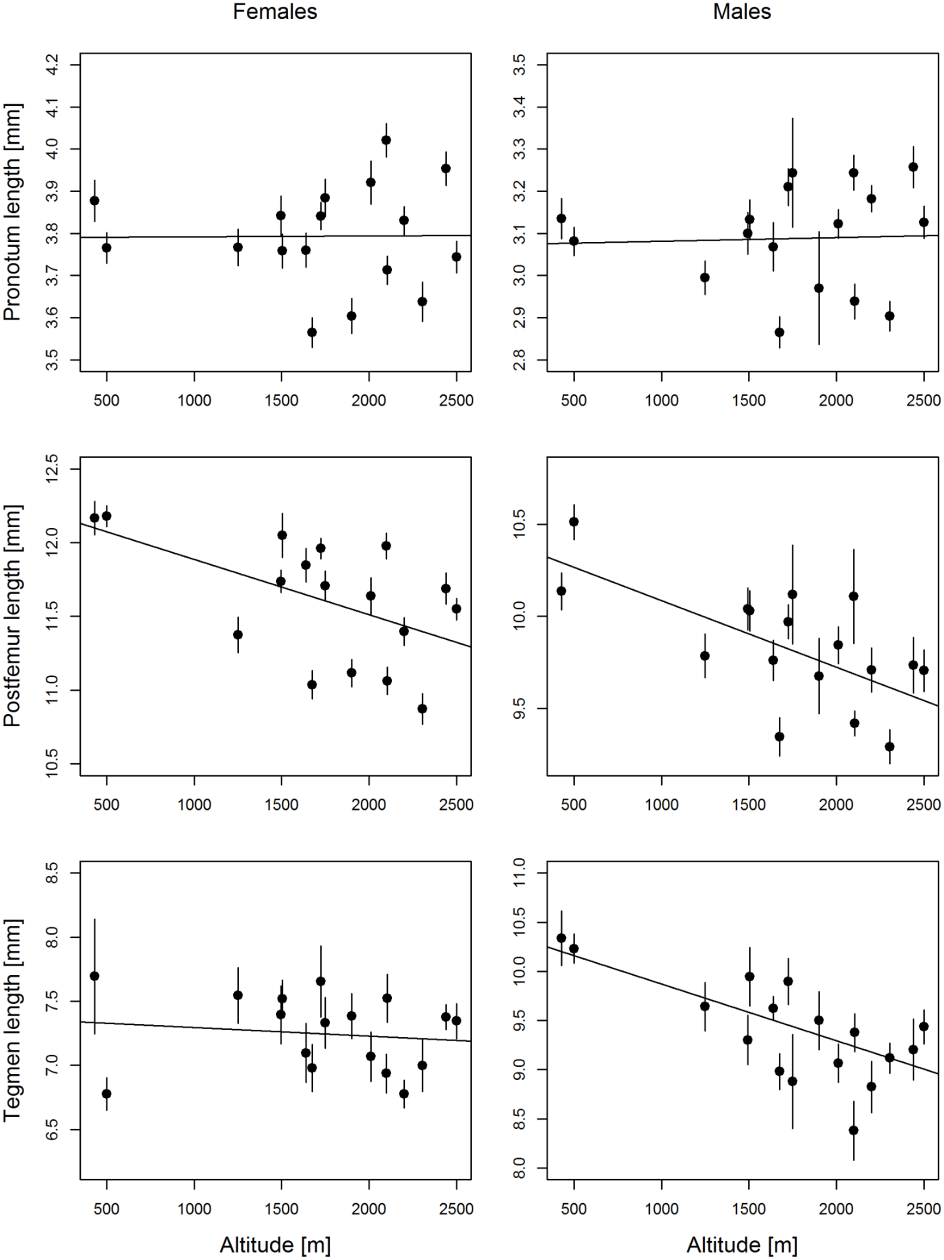
Altitudinal clines in morphology of *Pseudochorthippus parallelus*. Data show morphological trends in both sexes across 17 populations from the Swiss and Italian Alps. Estimates and their standard errors were independently for all populations while the regression lines were estimated using a mixed effects model fit.

Like most Gomphocerinae, *P*. *parallelus* exhibits a considerable sexual size dimorphism with substantial intrasexual phenotypic variability. Pronotum length ranged 3.2–4.4 mm in females and 2.4–3.5 mm in males, postfemur length 10.3–13.5 mm in females and 8.3–11.4 mm in males, and tegmen length 5.3–9.7 mm in females and 7.2–12.4 mm in males (Fig 2). A single female (0.2% of all individuals) from one lowland site at 430 m was macropterous with a tegmen length of 15.3 mm. This individual was excluded from the analysis of spatial variation in wing morphology. There was no significant temporal trend in any of the three traits in any of the sexes, except for a negative temporal trend in tegmen length of males (b = -0.022 ± 0.007, t_5.8_ = -3.02, P = 0.02, all others t < -1.79, P > 0.10).

Pronotum length did not change with altitude, neither in females (b = 0.001 ± 0.005, t_13.4_ = 0.18, P = 0.85) nor in males (b = 0.000 ± 0.005, t_12.3_ = 0.09, P = 0.92) (Fig 2). Postfemur length, in contrast, decreased significantly with altitude in both females (b = -0.041 ± 0.015, t_13.5_ = -2.71, P = 0.017) and males (b = -0.039 ± 0.009, t_10.3_ = -4.27, P = 0.0015) (Fig 2). In both cases there was no significant difference in slopes between females and males (pronotum: t_27.2_ = 0.079, P = 0.94, femur: t_27.4_ = 0.12, P = 0.91). Tegmen length, on the contrary, did not change with altitude in females (b = -0.007 ± 0.014, t_13.4_ = -0.48, P = 0.63), but decreased significantly in males (b = -0.060 ± 0.010, t_5.3_ = -6.31, P = 0.0012) (Fig 2), with the difference in slopes being significant (t_25.6_ = -3.50, P = 0.0017).

There was significant among-site variability (beyond the effect of altitude) for pronotum length in both sexes (females: R = 0.31 ± 0.09, χ^2^_1_ = 62.5, P < 10^−14^, males: R = 0.34 ± 0.13, χ ^2^_1_ = 36.4, P < 10^−9^), for postfemur length in both sexes (females: R = 0.37 ± 0.10, Χ^2^_1_ = 83.7, P < 10^−19^, males: R = 0.17 ± 0.08, χ ^2^_1_ = 9.0, P = 0.0013), and for tegmen length in females (females: R = 0.10 ± 0.01, χ ^2^_1_ = 11.4, P = 0.00036, males: R = 0.02 ± 0.03, χ ^2^_1_ = 0.00, P = 1.0).

### Colour polymorphism

All five colour morphs were represented in the collection: 15% of all individuals were completely green, 22.8% were green with brown postfemora (= 37.8% predominately green), 26.7% were brown dorsal stripe morphs, 0.5% were classified as green with brown sides (= 27.2% intermediate) and 35% as completely brown. There was no evidence that morphs differed in morphology (Table 2).

**Table 2:** Tests for morphological differences among colour morphs in *Pseudochorthippus parallelus*. Each row refers to independent tests from different linear model fit. The rarest morph (green with brown sides) with only 3 specimens in the sample was excluded from the analysis. F tests are based on linear mixed models that controlled for altitude and year of sampling as a fixed effect and for site identity as a random effect.

Sex	Trait	F	df	P
Female	Pronotum	1.39	3, 325.3	0.25
Female	Postfemur	0.39	3, 318.8	0.76
Female	Tegmina	0.37	3, 323.3	0.77
Male	Pronotum	0.75	3, 211.5	0.53
Male	Postfemur	0.90	3, 209.7	0.44
Male	Tegmina	0.34	3, 156.5	0.80

Morph composition was similar between the sexes for green morphs (females 17%, males 12%), dorsal stripe morphs (females 27%, males 26%), green with brown sides (females 0.6%, males 0.4%) and purely brown morphs (females 37%, males 31%). Only green with brown legs appeared somewhat more common among males (females 18%, males 30%). After controlling for the effect of altitude and year of sampling, there were non-significant trends for brown morphs to be less common (b = -0.86 ± 0.48, z = -1.79, P = 0.073) and green with brown legs to be more common in males (b = 0.64 ± 0.36, z = 1.76, P = 0.078), but clearly no significant differences with respect to green (b = -0.36 ± 0.43, z = 0.84, P = 0.40) or dorsal stripe morphs (b = -0.06 ± 0.29, z = -0.20, P = 0.84).

**Fig 3:**
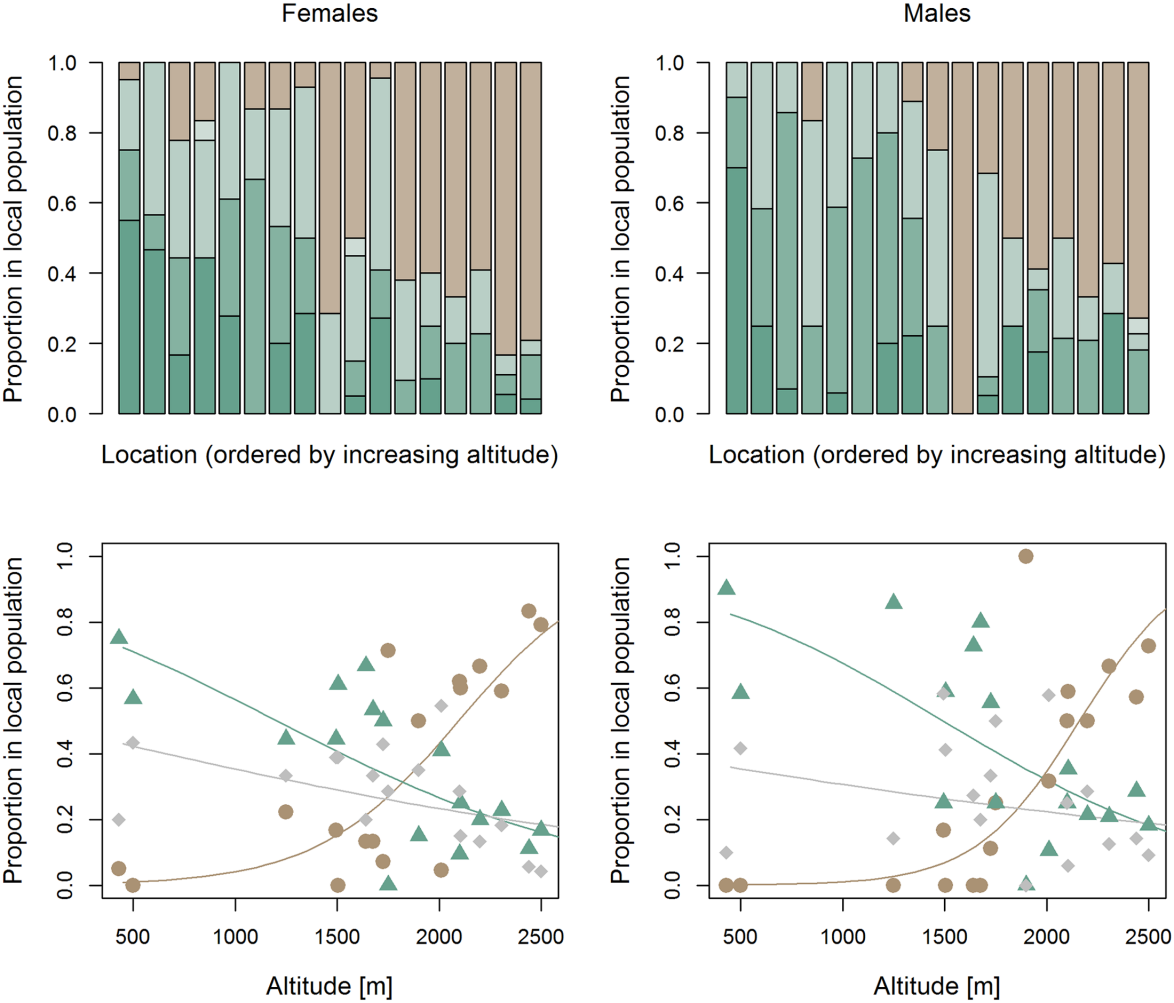
Altitudinal clines in colour morph composition of *Pseudochorthippus parallelus*. Data show colour morph trends across 17 populations from the Swiss and Italian Alps. Upper row: Complete distribution across all five colour morphs in both sexes (see Fig 1 for the colour code). Lower row: Clines for the pooled morphs predominately green (green triangles), predominately brown (brown circles) and intermediate (grey diamonds).

Colour morph composition changed significantly with altitude (Fig 3). Predominately green morphs significantly declined with increasing altitude in both sexes (females: b = -0.129 ± 0.029, z = 4.47, P < 10^−5^, males: b = -0.147 ± 0.039, z = -3.73, P = 0.00019), whereas predominately brown morphs increased (females: b = 0.316 ± 0.064, z = 4.88, P < 10^−5^, males: b = 0.409 ± 0.060, z = 6.76, P < 10^−10^). Intermediate morphs also tended to decline (females: b = -0.060 ± 0.028, z = -2.16, P = 0.031, males: b = -0.044 ± 0.039, z = -1.13, P = 0.26). In none of the cases were the slopes of females and males significantly different from each other (all |z| < 1.44, P > 0.14).

There was significant among site variability (beyond the effect of altitude and year of sampling) on the occurrence of predominately brown morphs in females, but not in males (females: R = 0.13 ± 0.05, χ ^2^_1_ = 1.7, P = 0.0001, males: R = 0.00 ± 0.01, χ ^2^_1_ = 0.0, P = 1.0) and significant spatial heterogeneity in the occurrence of predominately green morphs (females: R = 0.05 ± 0.03, χ ^2^_1_ = 3.12, P = 0.036, males: R = 0.09 ± 0.06, χ ^2^_1_ = 5.44, P = 0.0099).

## Discussion

We here report altitudinal clines in morphology and colour morph composition across 17 populations of the meadow grasshopper *P*. *parallelus* from the Swiss and Italian Alps. Pronotum length did not change neither in males nor in females, while postfemur length decreased with altitude in both sexes. Tegmen length decreased only in males, but not in females. This suggests that body size (as best quantified by pronotum length) did not vary across the altitudinal gradient (i.e. there is no evidence for altitudinal Bergmann or converse Bergmann clines), while shape changed with high-altitude populations having shorter appendices (following Allen’s rule). Furthermore, we found that green morphs, being prevalent at lower altitudes, progressively disappear at higher altitudes. At the same time, brown morphs, typically rare in the lowlands, increase in frequency − with a particularly steep increase between 1,500 m and 2,000 m asl − and become predominant at high altitudes. The colour pattern is remarkably consistent across the two sexes, suggesting shared colour-determining influences on females and males.

### Morphology

It was initially unexpected to find that altitudinal trends differed so markedly among the three morphological traits measured, with strong trends in postfemur length and a complete absence of trends in pronotum length in both sexes. The pronotum forms a distinct structural part of the body, while legs and wings represent appendices that vary independent of body size. Our data suggest that postfemur length partly conflates size with shape variation and that the inference of converse Bergmann’s rule based only on measurements of body appendices (such as postfemur length) in arthropods should be treated with some caution. Instead the pattern may suggest a conformance to Allen’s rather than converse Bergmann’s rule.

Allen’s rule has been initially described for endotherms [4] and the underlying causes may differ between endotherms and ectotherms in principle. But just like endotherms, grasshoppers may well lose heat via the wings or legs, so that the original reasoning of Allen’s rule may well apply. Indeed, conformance to Allen’s rule has been reported in other arthropods, including grasshoppers [5, 13]. However, besides its role in thermoregulation, there could be other advantages of more compact stature or constraints that prevent the development of longer legs/wings. For example, the shift in habitats with shorter overall height of alpine meadows compared to the long, lush lowland habitats may favour more compact build. In particular wind chill may be a strong factor at higher altitudes that either may affect individual performance through thermoregulation or manoeuvrability.

Interestingly, the trends in tegmen length were sex-specific. Wing length is highly sexually dimorphic in this species and a reduction in wing length at higher altitudes may only be relevant to the (longer winged) males. The tegmina are used in sound production in male grasshoppers and are therefore likely to be relevant to sexual selection [36–38]. If longer wings improve song attractiveness, sexual selection will favour longer wings, which may be possible to evolve in males at low altitudes, but sexual selection may be counteracted by opposing natural selection under harsh conditions at higher altitudes. In females, the tegmina are already reduced and adjoined with the abdomen, forming a compact body shape irrespective of altitude. This might have evolved for other reasons and selection for further reduction at high altitudes may not be as strong in females. Our data suggest that the balance between sexual and natural selection changed with altitude, with the relatively stronger natural selection at higher and relatively strong sexual selection at lower altitudes.

### Colour morph distribution

Our data illustrate the predominance of brown individuals in populations at altitudes above 2,000 m asl. Such brown populations of *P*. *parallelus* have occasionally been described as forma ´caffra´ [39] and at times also as a different species, *C*. *caffer*, [40], but the nowadays accepted treatment considers ´caffer´ as a colour morph of *P*. *parallelus* (see [24] for a review of the *parallelus*-group). This treatment is also supported by the absence of detectable morphological differences between morphs [41]. Increased frequencies of brown morphs at higher altitudes have been reported from other mountain ranges [42–45], while European lowland populations typically show less than 10% of brown individuals, even if it can be locally as high as 20–30% [19, 22, 44]. Our study now illustrates that the transition is remarkably steep between 1,500 and 2,000 m asl, suggesting a rather sharp change in the selective regime in this region (see [46] for a similarly sharp change at the same altitude in a lizard).

Following the reasoning of the thermal melanism hypothesis, we suspect that morph differences in thermoregulatory capacity might explain the sharp transition. Dark-coloured individuals heat up more rapidly at a given level of solar radiation [47], which is likely to be more critical at high altitudes, such that darkness of the body is associated with coolness of the habitat [7]. In line with this, dark morphs in several insects indeed increase in frequency at high altitudes [48, 47, 7]. Being overall darker than green individuals, brown individuals of *P*. *parallelus* may benefit from any difference in thermoregulatory capacity. The potential relevance of even slight differences in body colour is illustrated by another alpine grasshopper, in which nymphs developmentally darken under low radiation conditions as a putative response to increased need for more efficient body heating [49]. Alternative or additional factors may well contribute to the predominance of brown morphs at high altitude, for example the short vegetation period, local habitat structure or the presence and type of predators [50]. In any case, the remarkably parallel trends in males and females suggest that selection is largely concordant between the sexes (and also suggest that the underlying loci are not sex-linked).

### Conclusions

We describe altitudinal trends in morphology and body colouration of the grasshopper *P*. *parallelus*. While high-altitude populations do not show reduced body size, they possess shortened appendices and darker body colour. Both features may be explained by an increased need for thermoregulatory capacity under limiting conditions at high altitudes that can help to maintain a longer activity window and thereby allow survival at high altitudes.
